# Fighting liver fat

**DOI:** 10.1530/EC-20-0174

**Published:** 2020-07-02

**Authors:** David Koeckerling, Jeremy W Tomlinson, Jeremy F Cobbold

**Affiliations:** 1 Medical Sciences Division, University of Oxford, Oxford, UK; 2 Oxford Centre for Diabetes, Endocrinology & Metabolism, University of Oxford, Oxford, UK; 3 Oxford Liver Unit, NIHR Oxford Biomedical Research Centre, Oxford University Hospitals NHS Foundation Trust, John Radcliffe Hospital, Oxford, UK

**Keywords:** non-alcoholic fatty liver disease

## Abstract

Non-alcoholic fatty liver disease is a chronic liver disease which is closely associated with components of the metabolic syndrome. Its high clinical burden results from the growing prevalence, inherent cardiometabolic risk and potential of progressing to cirrhosis. Patients with non-alcoholic fatty liver disease show variable rates of disease progression through a histological spectrum ranging from steatosis to steatohepatitis with or without fibrosis. The presence and severity of fibrosis are the most important prognostic factors in non-alcoholic fatty liver disease. This necessitates risk stratification of patients by fibrosis stage using combinations of non-invasive methods, such as composite scoring systems and/or transient elastography. A multidisciplinary approach to treatment is advised, centred on amelioration of cardiometabolic risk through lifestyle and pharmacological interventions. Despite the current lack of licensed, liver-targeted pharmacotherapy, several promising agents are undergoing late-phase clinical trials to complement standard management in patients with advanced disease. This review summarises the current concepts in diagnosis and disease progression of non-alcoholic liver disease, focusing on pragmatic approaches to risk assessment and management in both primary and secondary care settings.

## Why fight liver fat?

### What is NAFLD?

Non-alcoholic fatty liver disease (NAFLD) is recognised as the most common aetiology of chronic liver disease, with an estimated global prevalence of 25.2%, and as a major cause of cirrhosis and hepatocellular carcinoma (HCC), projected to become the leading indication for liver transplantation during this decade (1). NAFLD is defined as the accumulation of liver fat (exceeding 5% of hepatocytes) without evidence for coexisting hepatic insults, namely viral or autoimmune hepatitis, use of steatogenic medication, or significant alcohol intake (2). Regarded as the hepatic manifestation of the metabolic syndrome in view of its intimate association with insulin resistance, obesity, hypertension and dyslipidaemia, NAFLD is a multi-system disease encompassing a histopathological spectrum of severity. This heterogeneous continuum ranges from simple, isolated steatosis (non-alcoholic fatty liver (NAFL)) to steatohepatitis with evidence of hepatocyte injury and necroinflammation (non-alcoholic steatohepatitis (NASH)) with or without hepatic fibrosis. In its advanced stages, NAFLD may progress to cirrhosis and its complications, including HCC. Its mounting prevalence and potentially aggressive nature combined with diagnostic and therapeutic barriers make NAFLD an important public health concern of the 21st century (3). Mirroring the obesity pandemic, its clinical and economic burden is reaching enormous proportions: 52 million people are estimated to suffer from NAFLD in Germany, France, Italy and the UK, incurring annual direct medical costs of €35 billion (2, 3).

The heterogeneity of disease progression is reflected in the multi-faceted pathogenesis of NAFLD. Hepatocellular fat accumulation arises when lipid export or degradation is exceeded by lipid import or synthesis. Main sources of hepatic lipid aggregation are a flux of free fatty acids (FFA) from peripheral adipose tissue (59%), followed by hepatic *de novo* lipogenesis (26%) and dietary intake (14%) (4). Traditionally, steatosis severity is graded according to the extent of triglyceride accumulation despite the recognition that, in general, triglycerides *per se* do not cause hepatocyte injury. In contrast, triglyceride accumulation appears to be an adaptive mechanism minimising hepatocyte injury from lipotoxicity caused by reactive lipids and fatty acids, such as cholesterol, FFAs, oxysterols or phospholipids. In chronic nutrient surplus, the ability or inability of the liver to compensate for fatty acid exposure by synthesising triglycerides determines whether lipotoxicity ensues. If compensatory mechanisms are overwhelmed, lipotoxicity originates from the generation of reactive oxygen species and dysfunction of unfolded protein responses. Hepatocytes exposed to chronic lipotoxicity initiate dysregulated regenerative processes which perpetuate inflammatory and fibrogenic stimuli (4, 5, 6, 7, 8). In normal homeostasis, insulin potently inhibits adipose tissue lipolysis. Insulin resistance, a fundamental characteristic of NAFLD, manipulates hepatic lipid metabolism and exacerbates adipocyte dysfunction, encouraging intrahepatic lipogenesis and fatty acid influx (7). Gut-liver axis dysfunction has been implicated in NAFLD pathogenesis through mechanisms which include generation of short-chain fatty acids, alterations in intestinal permeability and bacterial translocation into the portal vasculature (5, 6, 7). Reductions in microbiome quality, quantity and diversity are documented in NAFLD, yet causality between ‘dysbiosis’ resolution and NAFLD improvement is not established (5).

### Natural history of NAFLD

The complex phenotype and variable progression rate of NAFLD reflect the overlapping influences of genetics, diet, comorbidities and metabolic discrepancies between individuals. A minority of patients advances to significant fibrosis, yet ambiguity exists regarding long-term outcomes and histological progression of NAFLD (9). Epidemiologically, global NAFLD prevalence was estimated at 25.24%, with highest and lowest prevalence rates in the Middle East (32%) and Africa (14%), respectively. Comparable estimates were reported from Europe (23.7%) and the US (24.1%) (1). NAFLD prevalence increases analogously with burgeoning obesity, T2DM, hyperlipidaemia and hypertension rates, doubling from 5.5% in 1980 to 11% in 2008 in the US (1, 10). Over the last decade, the frequency of NAFLD as an indication for liver transplantation surged by 170% and HCC cases attributable to NAFLD simultaneously increased from 8.2% to 13.5% with NAFLD on trajectory to becoming the most common indication for liver transplantation during this decade (11).

While mortality data in NAFLD is difficult to interpret owing to discrepancies in the design of studies assessing survival, robust evidence indicates that fibrosis stage is the most relevant prognostic marker in NAFLD. Early mortality data was summarised by a meta-analysis demonstrating higher all-cause mortality for NAFLD patients compared to the general population (OR 1.57, 95% CI: 1.18–2.10, *P* = 0.002) (12). The predominant causes of death identified were cardiovascular complications, malignancy and liver-related complications in descending order (13). One of the longest follow-up studies available (mean follow-up 26.4 years) supports the notion that NAFLD confers increased all-cause mortality (HR 1.29) with advanced fibrosis being the only histological parameter to predict mortality (14). Hepatic fibrosis in NAFLD is categorised relative to location and extent: stage 1 (F1) is defined as perisinusoidal fibrosis alone, stage 2 (F2) as perisinusoidal plus periportal fibrosis, stage 3 (F3) includes bridging fibrosis, and stage 4 (F4) is cirrhosis (15, 16). Angulo *et al.* analysed 619 patients with biopsy-proven NAFLD retrospectively, validating fibrosis stage as the most reliable histological characteristic to predict adverse outcomes (17). A recent meta-analysis with 17,000 patient-years follow-up substantiated these findings. All-cause mortality progressively heightened with each subsequent fibrosis stage (mortality rate ratios by fibrosis stage: F1, 1.58; F2, 2.52; F3, 3.48; F4, 6.44) and liver-related mortality grew exponentially with fibrosis progression (F1, 1.41, F2, 9.57; F3, 16.69; F4, 42.30) (18). In the largest paired biopsy study to date (*n* = 646, mean follow-up 20 years) fibrosis stage alone independently predicted liver-related morbidity and overall mortality, whereas the presence of NASH had no significant bearing on outcomes (19).

Unsurprisingly given the cardiometabolic risk factors inherent to the metabolic syndrome, cardiovascular disease is the most common cause of death in NAFLD (17, 19, 20). The intricate association with the metabolic syndrome complicates the distinction between NAFLD as an independent phenomenon in cardiometabolic disease pathogenesis and NAFLD as a bystander sharing common aetiological foundations with cardiometabolic disease (20). Epidemiological evidence links NAFLD to cerebrovascular, coronary and peripheral vascular disease, as well as to subclinical artery intima-media thickness and arterial wall stiffness (20). NAFLD is also closely associated with chronic kidney disease and early renal dysfunction with microalbuminuria (20). NAFLD and T2DM coexistence is common, promoting adverse outcomes in a synergistic and bi-directional manner. NAFLD is present in up to 70% of patients with T2DM, while NAFLD patients exhibit five-fold increased risks of T2DM development (21). T2DM and NAFLD coexistence is believed to drive micro- and macrovascular complications in T2DM and accelerate fibrogenesis in NAFLD (21). Insulin resistance is a cardinal feature of T2DM, cardiovascular disease and NAFLD pathogenesis, thereby potentially confounding the relationship between NAFLD and cardiometabolic outcomes (22).

Historically, NAFL was viewed as a benign disease state and NASH as its aggressive counterpart. Current evidence suggests that neither NAFL nor NASH, but only the presence of fibrosis directly influences clinically relevant outcomes. Do NAFL and NASH differ in their fibrogenic potential? In a systematic review of 221 patients with biopsy-proven NASH, 37.6% of patients demonstrated fibrosis progression over a 5.3 years mean follow-up (23). The indolent nature of NAFL was questioned by several small-scale longitudinal studies, observing fibrosis progression in 24–61% of NAFL patients with some individuals even reaching advanced fibrosis and end-stage liver disease (24, 25, 26, 27). In a meta-analysis of 411 NAFLD patients, 33.6% experienced fibrosis progression, 43.1% had static fibrosis stage, and 22.3% demonstrated fibrosis regression. This pooled analysis indicates that NAFLD is not always progressive, yet both NAFL and NASH appear to hold fibrogenic potential. If fibrosis progresses, however, it occurs approximately twice as quickly in NASH with an annual fibrosis progression rate of 0.14 stages compared to 0.07 stages in NAFL (28). Independent of NAFL/NASH status, 20% of patients with fibrosis progression were classified as ‘rapid progressors’ with the evolution from no fibrosis to advanced fibrosis in over just 6 years. Presently, no reliable methods exist to identify this high-risk subset of patients, although certain clinical features, especially obesity and T2DM, seem to confer increased risks of fibrosis progression, as detailed subsequently (28).

## Choosing your battles

### Establishing the diagnosis

In the absence of public screening programmes, NAFLD should be suspected in metabolically predisposed patients presenting with asymptomatically elevated aminotransferases or incidentally detected liver fat on abdominal imaging, prompting a comprehensive workup of all components of the metabolic syndrome and systematic exclusion of competing aetiologies of liver dysfunction (Table 1) (29). If symptoms are present, they are of non-specific nature, such as fatigue, mild right upper quadrant pain or epigastric fullness (8). NAFLD tends to remain asymptomatic until progression to end-stage liver disease and decompensation with ascites, hepatic encephalopathy and variceal haemorrhage occurs. While hepatomegaly and central adiposity are frequent, there are no pathognomonic examination findings in NAFLD. Rarer findings include acanthosis nigricans in insulin resistance and dorsocervical lipohypertrophy in NASH (8, 30). Since the prevalence of NAFLD in those with T2DM and obesity is substantial and most patients with NAFLD have normal liver biochemistry (1), there is an argument for suspecting NAFLD in all such patients and conducting risk stratification, although data to support such an approach is still being collated.

**Table 1:** Comprehensive assessment in suspected NAFLD.

Comprehensive assessment in suspected NAFLD			
Metabolic work-up		Exclude secondary hepatic insults	
Type 2 diabetes mellitus	Fasting blood glucose, HbA1c, oral glucose tolerance test	Alcohol excess	>20 g/day (women) and >30 g/day (men), AST:ALT ratio
Obesity	BMI, waist circumference, change in weight	Steatogenic medication	Amiodarone, diltiazem, steroids, valproic acid, tamoxifen, anti-psychotics, highly active retroviral therapy
Hypertension	Repeated blood pressure monitoring	Rare causes of hepatic steatosis	Refeeding syndrome, lipodystrophy, total parenteral nutrition
Dyslipidaemia	Serum total and HDL cholesterol	Hepatitis B/C infection	Viral hepatitis serology
Endocrine disorders	Hormonal profiling for hypothyroidism, hypogonadism, hypopituitarism and polycystic ovarian syndrome	Rare chronic liver diseases	Haemochromatosis (ferritin and transferrin saturation), Wilsons disease (caeruloplasmin), autoimmune hepatitis (immunoglobulins), alpha-1-antitrypsin deficiency (alpha-1-antitrypsin levels)

ALT, alanine aminotransferase; AST, aspartate aminotransferase; HbA1c, haemoglobin A1c; HDL, high-density lipoprotein; NAFLD, non-alcoholic fatty liver disease.

Initially, appropriate history taking should identify other causes of hepatic steatosis, including steatogenic medications (31), refeeding syndrome, total parenteral nutrition and lipodystrophy (32). Although excess alcohol intake requires exclusion as a common aetiology for liver steatosis, this is often difficult in clinical reality. In NAFLD, alanine aminotransferase (ALT) classically exceeds aspartate aminotransferase (AST) levels, while alcoholic liver disease frequently demonstrates AST:ALT ratios of >1.5 (32). With progressive fibrosis in NAFLD, however, AST levels may proportionally rise, increasing the AST:ALT ratio (32). NAFLD is the most common reason for raised transaminases (11), yet up to 78% of NAFLD patients exhibit non-elevated liver enzymes (1, 33), and even advanced disease often exists despite normal liver function tests (9). In fact, the entire histological continuum of NAFLD was observed in patients with normal liver biochemistry (34). This illustrates the imperfect nature of transaminases as prognostic and diagnostic tools in NAFLD, emphasising that clinicians should not rely upon transaminases to establish NAFLD diagnosis. Coexisting insults causing hepatic dysfunction should be ruled out through extended liver testing (Table 1) (35). Aberrations in serum levels of thyroid, sex and growth hormones are known to contribute to the development of metabolic syndrome and NAFLD, necessitating extended hormonal profiling if there is a high index of clinical suspicion for endocrine abnormalities (36).

Once NAFLD is suspected, evidence of hepatic steatosis is required to satisfy diagnostic criteria. Quantification of hepatic steatosis is prognostically insignificant, and steatosis often regresses as fibrosis progresses (4, 37). Abdominal ultrasound is the most accessible imaging modality for steatosis assessment and benefits from its non-invasiveness, low cost and absence of radiation. Ultrasound is sensitive (85%) and specific (95%) for detecting moderate to severe steatosis (>33% steatotic hepatocytes); however, its sensitivity deteriorates when <30% of hepatocytes are affected (38, 39). Increased echogenicity, the characteristic ultrasound finding in hepatic steatosis, is also present in fibrosis and early cirrhosis, reducing the reliability of ultrasound in coexisting liver disease aetiologies (8, 37, 39). To overcome the limitations of traditional ultrasound in diagnosing mild steatosis, steatosis-specific imaging methods were developed. The controlled attenuation parameter (CAP) measures ultrasound attenuation by hepatic lipid content, demonstrating improved accuracy in detecting mild steatosis (steatosis >10% – AUROC 0.91) (37, 39, 40). Magnetic resonance-based imaging technologies measuring the proton density fat fraction (PDFF) are considered the gold standard for non-invasive steatosis assessment, enabling hepatic fat mapping with extremely high accuracy (AUROC 0.99). Magnetic resonance spectroscopy (MRS) assesses PDFF directly through differences in water and lipid peaks on resonance frequency domains, whereas MRI indirectly estimates water and fat content through time-dependent oscillations in MR signals (41). Due to its direct nature of measurement, potentially higher accuracy is ascribed to MRS, although MRI-PDFF demonstrated comparable operating performances across various studies (41). While MRI-PDFF permits steatosis quantification throughout the liver, MRS-PDFF evaluates single voxels of hepatic parenchyma, incurring risks of sampling bias. Both techniques are confined to research settings given the expense, infrastructure and expertise required for their acquisition and interpretation (37, 41).

Identifying those at greatest risk of disease progression or with already advanced disease is essential to efficiently target therapeutic interventions. As the current reference standard for diagnosis, prognosis and treatment response assessment in NAFLD, clinical trials rely heavily on liver biopsy, supplying investigators with a wealth of histological information about liver architecture, presence and extent of steatosis, necroinflammation and fibrosis (42). However, liver tissue acquired by biopsy represents merely 1/50,000 of total liver volume and incurs significant risks of underestimating disease severity due to spatial sampling variability and non-uniform disease distribution (37, 39). Liver biopsies are expensive, invasive and prone to interobserver variability (37). The substantial epidemiological burden of NAFLD necessitates the implementation of more pragmatic approaches to diagnosis and risk stratification in clinical practice.

### Risk stratification

Given that fibrosis is the strongest predictor of outcome, risk stratification based on fibrosis severity can determine who would benefit from liver-directed therapeutic interventions in specialist secondary care services. Certain populations of patients experience increased risks of NAFLD progression to advanced fibrosis. In comparison to pre-menopausal women, males and post-menopausal females displayed higher fibrosis stages with onset and duration of menopause as independent risk factors for fibrosis presence (43). The relationship between ethnicity and NAFLD severity is complex. While NAFLD prevalence is disproportionally lower among African American patients and higher among Hispanic patients compared to white populations, these discrepancies are less marked in high-risk cohorts (T2DM and obesity) and the rates of advanced fibrosis do not seem to differ significantly between ethnicities (44). This indicates that ethnicity may play a comparatively greater role in determining NAFLD prevalence rather than severity (45). Genetic susceptibility to NAFLD and its progression was first established by genome-wide association studies suggesting that ethnic disparities in NAFLD prevalence are, in part, dependent on patatin-like phospholipase domain-containing protein 3 (PNPLA3) genotypes (46, 47). Subsequent publications strengthened links between PNPLA3 polymorphisms, NAFLD susceptibility and advanced histology (47). Additional risk alleles such as TM6SF2 were since identified (48). Despite their common prevalence, these polymorphisms explain minor proportions of disease phenotype variability and are currently unsuitable for population-based risk assessment (47). In cross-sectional studies, age consistently predicted advanced fibrosis, yet this fails to persevere in longitudinal studies, implying that age reflects cumulative exposures to metabolic insults and disease duration rather than fibrosis progression rate (28, 43). Extensive evidence identified obese and diabetic patients as high-risk groups for fibrosis progression (14, 17, 25, 26, 28, 43). Accordingly, deterioration and improvement in fibrosis correlate with chronological weight gain or loss, respectively, while T2DM and NAFLD coexistence accelerates fibrogenesis (21, 43, 49, 50). Early histological data suggested that the risk of NASH could approach 40% in T2DM (50, 51). In a cross-sectional study of 1900 diabetic patients, the prevalence of advanced fibrosis was as high as 17.7% (50, 52). In two recent histological studies investigating patients with concomitant T2DM and NAFLD, advanced fibrosis even reached 40–41% prevalence (50, 53, 54).

There are many examples of non-invasive approaches to risk stratification in NAFLD including simple composite scoring systems, transient elastography or specialist panels for biomarkers of extracellular matrix remodelling. Simple scoring systems indirectly measure fibrogenesis and are derived from clinical risk factors of fibrosis progression (e.g. obesity, T2DM) and routinely available biochemistry reflecting liver dysfunction (AST, ALT, platelet count, albumin, etc). The NAFLD fibrosis score (NFS) and the Fibrosis-4 index (FIB-4) are two extensively validated, simple scoring systems with high negative predictive values for the exclusion of advanced fibrosis. The NFS (composed of BMI, age, presence of T2DM, AST:ALT ratio, platelet count and albumin) was specifically constructed and validated in biopsy-proven NAFLD (34, 37, 55), whereas the FIB-4 algorithm (composed of AST, ALT, platelet count and age) was originally derived for use in hepatitis C/HIV coinfection and later effectively validated in NAFLD (35, 56). Both models utilise dual diagnostic cut-off values to exclude or diagnose advanced fibrosis. The NFS excludes advanced fibrosis with a negative predictive value (NPV) of 88–93% and detects advanced fibrosis with a positive predictive value (PPV) of 82–90% (35, 55). The FIB-4 reliably excludes advanced fibrosis (NPV 90–95%) and diagnoses advanced fibrosis with moderate accuracy (PPV 80%) (35, 56). These simple models generally demonstrate inferior accuracy in discriminating between fibrosis stages compared to direct measures of fibrogenesis, yet their high negative likelihood ratios and accessibility make them valuable screening tools to exclude advanced fibrosis. While the NFS is more extensively validated, the FIB-4 performs slightly better in head-to-head comparisons and requires fewer variables for calculation, making it suitable for triaging NAFLD patients in primary care settings (8, 34, 35). Both models generate indeterminate scores in a considerable proportion of cases (40–50%) necessitating a two-tiered system for risk stratification (34). Depending on local accessibility and expertise, the second tier should involve transient elastography or proprietary panels which directly measures biomarkers of fibrogenesis/fibrinolysis.

One such commercial panel is the Enhanced Liver Fibrosis (ELF™) Test which incorporates three fibrosis biomarkers: hyaluronic acid, tissue inhibitor of metalloproteinase-1 and amino-terminal propeptide of type III procollagen. ELF was validated in biopsy-proven NAFLD, exhibiting marginally improved accuracy in predicting advanced fibrosis for adult (AUROC 0.93) and paediatric (AUROC 0.99) patients compared to non-proprietary algorithms (37, 57, 58). Its single diagnostic cut-off value for advanced fibrosis precludes indeterminate results; however, increased cost compared to non-commercial systems rationalise its position as a second-line risk stratification approach (34, 35).

Transient elastography (TE) technologies, such as FibroScan, are validated for hepatic fibrosis assessment in various liver disease aetiologies, including NAFLD (59). TE evaluates liver elasticity by measuring shear wave velocity, relying on the principle that hepatic parenchyma gradually loses elasticity with cumulative fibrotic tissue deposition. TE is a quick, painless and non-invasive procedure that assesses 1/500 of total liver volume (1/50,000 for liver biopsy), ameliorating sampling bias (60). TE capably excludes advanced fibrosis with few false negatives and demonstrates superior accuracy in identifying advanced fibrosis (AUROC 0.93–0.95) compared to simple scores (35, 59). Pitfalls of TE include the lack of universally validated, diagnostic cut-off values for individual fibrosis stages and its considerable failure rate in clinical practice, especially with obese individuals (60). In a prospective study of 13,000 TE examinations, failure rate ranged from 1% in lean patients to 42% in morbidly obese patients with an additional 16% unreliable readings, making nearly one-fifth of all readings uninterpretable (61). Failure rates were reduced by the introduction of XL probes which cater towards obese patients (35, 37, 60).

**Figure 1:**
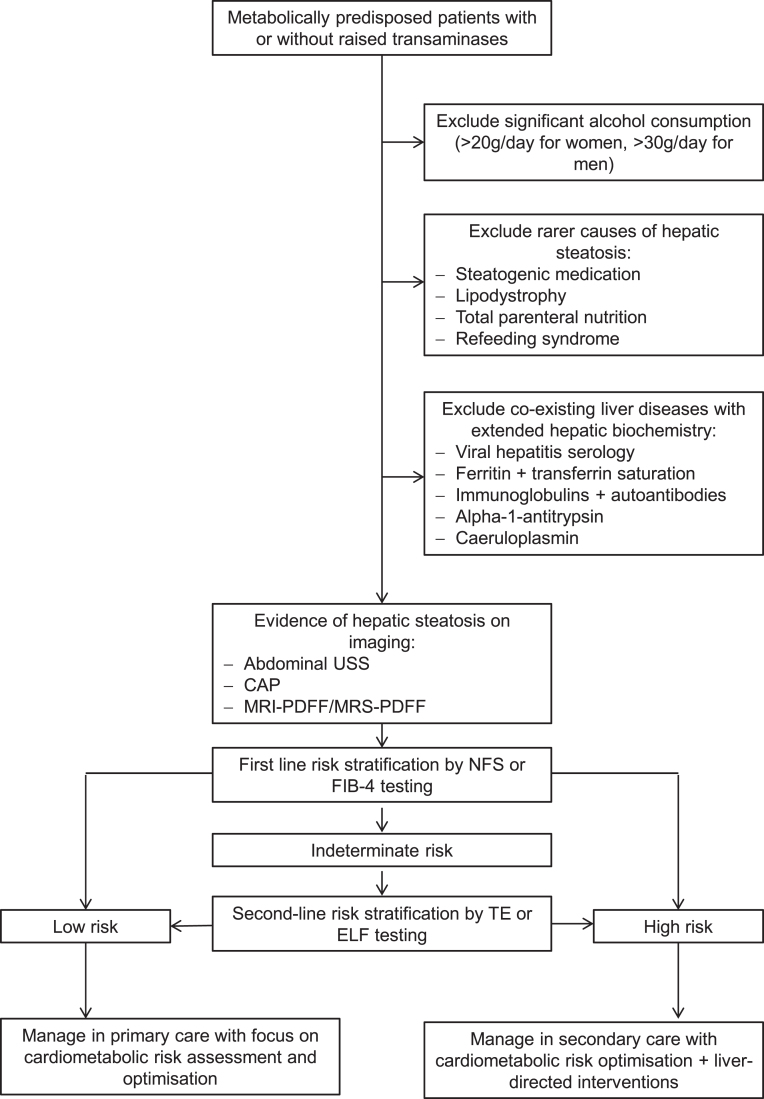
Suggested risk stratification algorithm in NAFLD. CAP, controlled attenuation parameter; ELF, enhanced liver fibrosis test; FIB-4, fibrosis-4 index; MRS, magnetic resonance spectroscopy; NFS, NAFLD fibrosis score; PDFF, protein density fat fraction; TE, transient elastography.

ELF or TE testing is recommended in the event of indeterminate outcomes from first-line non-commercial models (FIB-4, NFS) to classify those patients in the ‘grey zone’ (45). If test result discordance or diagnostic uncertainty persists after repeated non-invasive testing, consideration of liver biopsy is appropriate (37, 60). NAFLD patients categorised as low risk for significant hepatic fibrosis can be managed in primary care with therapeutic focus on cardiometabolic risk optimisation, since the probability of adverse liver-related outcomes within a 10- to 15-year time frame is low (28, 62). High-risk patients warrant specialist hepatology referral for liver-focused management in addition to cardiometabolic risk factor control in secondary care settings (Fig. 1). Cost-effectiveness analyses of this two-tiered risk stratification approach demonstrated five-fold increased detection rates of advanced fibrosis and cirrhosis while incurring specialist referral rates of 10% and reducing unnecessary referrals by 81% with significant cost savings (34, 42, 63).

## Deploying your forces

### The multidisciplinary approach

The multisystemic nature of NAFLD necessitates a holistic management approach with a varying focus on hepatic and cardiometabolic risk control depending on the disease stage. A purely hepato-centric treatment approach would be unsatisfactory given that cardiovascular disease is the principal cause of mortality in NAFLD (~40%) with malignant and non-malignant liver diseases accounting for 10% of deaths (45). Integration of multidisciplinary pathways across primary and secondary care is required to achieve NAFLD diagnosis and risk assessment, therapeutic optimisation of cardiometabolic risk and diabetic control, adjustment of lifestyle and diet, as well as the initiation of liver-directed interventions and recruitment into clinical trials (45). The ideal multidisciplinary team would include expertise in hepatology, diabetology/endocrinology and/or metabolic medicine, dietetics, lifestyle advice and weight management across primary and secondary care; however, resource limitations frequently hinder the completeness of multidisciplinary teams (64). Holistic, multidisciplinary management is widely advocated in NAFLD, yet few studies evaluated its real-world effectiveness and data favouring its utility remain scarce. Moolla *et al.* prospectively followed NAFLD patients attending a dedicated, multidisciplinary metabolic hepatology clinic in Oxford, UK, finding considerable improvements in liver and cardiometabolic health with reductions in ALT, weight, HbA1c, total cholesterol, QRisk3 score, and liver stiffness measurements (65).

### Optimisation of cardiometabolic risk

Cardiometabolic interventions in NAFLD are founded on the central hypothesis that reversal of insulin resistance and hyperglycaemia alleviates cardiometabolic risk while simultaneously decelerating steatohepatitis activity and fibrosis (22). Independent of liver-related risk status and healthcare setting, lifestyle interventions targeting weight, diet and overall fitness remain the cornerstone of therapy for all NAFLD patients (39, 62, 66). The incremental effect of weight loss on histological improvement is well documented; greater and more sustained weight loss correlating with more substantial histological improvements. Amelioration of ALT levels, steatosis and NASH is seen even with modest weight loss (>5%), while NASH resolution and fibrosis regression were observed in higher degrees of weight reduction (≥10%) (62, 64, 67, 68). A serial biopsy study (*n* = 293) found NASH resolution in 90% and fibrosis regression in 45% of patients who achieved ≥10% total body weight loss (68). However, the long-term effectiveness of lifestyle-based weight loss interventions remains unclear, since these modifications are difficult to sustain. For example, only one in ten participants of the aforementioned study lost ≥10% total body weight and the vast majority (70%) did not achieve even ≥5% weight loss. Carefully selected patients at low risk of hepatic decompensation and unresponsive to lifestyle interventions may benefit greatly from bariatric surgery, given its substantial impact on body weight and obesity-related comorbidities (45, 62). Roux-en-Y gastric bypass surgery remains the most effective treatment for obesity as it induces greater, more sustained weight loss than other procedures with almost immediate, weight-independent effects on glycaemic control, insulin sensitivity and GLP-1 secretion (45, 69). Sleeve gastrectomy produces less marked, but comparable, effects to Roux-en-Y bypass surgery without significantly altering upper gastrointestinal tract anatomy (45, 62, 69). In a recent pooled analysis of 32 cohort studies, bariatric surgery resolved steatosis in 66%, inflammation in 50%, ballooning degeneration in 76% and fibrosis in 40% of patients (70). As NAFLD is not a primary indication for bariatric surgery and no randomised controlled trials exist in this field, these data cannot be regarded as level 1 evidence (62). Furthermore, 12% of patients experienced worsening of NAFLD features following bariatric surgery (70). Mechanisms underpinning this deterioration are unknown, but were proposed to be related to the type of bariatric operation employed (45). No studies to date found reductions in liver-related mortality after bariatric surgery, underscoring the need for long-term, controlled trials in this area (69).

The role of dietary composition in NAFLD development and management is under extensive investigation. The ideal diet for NAFLD patients is yet to be established. Diets high in polyunsaturated fatty acids (PUFA) exhibited beneficial effects upon insulin sensitivity, visceral adiposity, hepatic triglyceride content and steatohepatitis independent of weight reduction (64, 71). Two randomised trials compared isocaloric diets enriched in PUFAs and saturated fatty acids (SFA), finding that SFAs promote visceral and hepatic lipid storage, whereas PUFAs generated a three-fold increase in lean tissue (72, 73). Being rich in PUFAs, the potential liver-specific benefits of the Mediterranean diet were explored, after previously demonstrating protective effects on cardiovascular disease and diabetes risk – two highly relevant outcomes in NAFLD (64). In a 6-week crossover study, the Mediterranean diet improved hepatic steatosis and insulin sensitivity independent of weight loss compared to an isocaloric low-fat, high-carbohydrate diet (74). Calorie vs carbohydrate restriction was compared in one short-term dietary intervention study which favoured carbohydrate restriction for reducing hepatic steatosis despite similar weight loss profiles between groups (75). Isotope labelling studies highlighted the role of refined sugar intake (particularly fructose from industrial rather than fruit-derived sources) in hepatic lipotoxicity by facilitating *de novo* lipogenesis, depleting hepatic ATP and generating uric acid (6). Daily refined sugar intake was linked to lower steatosis grade but higher fibrosis stage (76). Fructose and refined sugar consumption should form part of thorough history taking in NAFLD and should be addressed accordingly (64).

Physical activity and exercise are strongly advocated for patients with NAFLD, although the evidence underpinning this management approach remains limited. Controlled studies with sufficient statistical power to outline exercise programmes or physical activity guidelines tailored to NAFLD patients are lacking (64). Mechanisms underlying proposed benefits of exercise in NAFLD pertain to improvements in peripheral insulin resistance, independent of weight loss (77). Pooled data from small-scale trials comparing aerobic and resistance exercise suggest that exercise in isolation (without weight loss) can generate relative reductions in hepatic lipid content by 20–30% (78). Variable forms of exercise (resistance, aerobic or high-intensity intermittent exercise) appear to have comparable impacts on hepatic steatosis (64, 78). Although emergent data indicate clinically meaningful benefits on steatosis reduction through exercise alone (20–30%), this improvement is modest compared to weight loss which can generate ≥80% reductions in liver fat content (64, 79). Combined approaches integrating lifestyle-based interventions for weight management, diet and exercise with realistic, achievable goals and regular follow-up are advised.

### Diabetes control in NAFLD

T2DM and NAFLD coexistence is a high-risk combination which synergistically accelerates morbidity, making optimisation of diabetic control imperative. The ideal anti-diabetic agent in NAFLD combines weight-reducing efficacy, cardiovascular event prevention, glycaemic control and cost-effectiveness while providing additional protective effects on liver histology (80).

The biguanide metformin is the first-line pharmacological agent for T2DM therapy and displays favourable effects on total body fat and insulin sensitivity. In a meta-analysis of 671 patients with NAFLD and T2DM, metformin failed to impact liver histology significantly despite reductions in HbA1c and weight (21, 81). Metformin does not appear to offer unique benefits in NAFLD and is not licensed for its treatment outside the context of diabetic control (21, 22).

Thiazolidinediones, selective ligands to peroxisome-proliferator-activated gamma receptors (PPAR gamma), act as insulin sensitisers by targeting adipocyte differentiation (50). In randomised, placebo-controlled trials (RCT), pioglitazone administration alleviated all histological characteristics of NASH (steatosis, inflammation, Mallory–Denk bodies and hepatocellular ballooning) in diabetic and non-diabetic patients (82, 83, 84). Although significant effects on fibrosis were not evident in individual trials, a pooled analysis of RCTs indicated that pioglitazone therapy led to fibrosis regression (85). Outside its potential liver-centric benefits, pioglitazone decreased cardiovascular event incidence in patients with T2DM and outperformed other anti-diabetic medications with regards to mortality-rate reductions in a European cohort (22, 86). Concerns about extensive adverse effects (increased risk of fractures, fluid retention, bladder cancer and weight gain) currently preclude the wider use of pioglitazone in patients with T2DM and NAFLD, although the evidence supporting these adverse events is disputed (21, 62).

Dipeptidyl peptidase-4 (DPP-4) inhibitors increase incretin availability and are widely employed as adjunctive oral therapy in T2DM (21). DPP-4 inhibitors have neutral effects on cardiovascular outcomes in T2DM with no evidence for additional benefits of clinical relevance in NAFLD, although adequately powered, controlled studies with primary histological endpoints are lacking (21, 22).

Incretin mimetics, particularly glucagon-like peptide-1 (GLP-1) receptor agonists, displayed attractive therapeutic potential in NAFLD and coexisting obesity and/or T2DM. GLP-1 agonists primarily stimulate glucose-dependent insulin secretion with important extra-pancreatic effects relating to satiety and insulin sensitivity (21, 50), thus improving glycaemic control and promoting weight loss via CNS modulation (21, 87). Meta-analysis of six phase III RCTs found that higher dose liraglutide (1.8 mg) significantly decreased ALT and hepatic steatosis levels, an effect intimately related to the magnitude of weight reduction (87, 88). In the landmark proof-of-concept LEAN trial (22, 87), liraglutide administration led to NASH resolution in a higher proportion of patients (39% vs 9%) and to fibrosis progression in a lower proportion of patients (9% vs 36%) compared with placebo (89). Whether weight loss exclusively lies at the heart of the mechanism underpinning phenotypic improvements needs to be clarified, although rodent studies suggest that these effects may be partly mediated by GLP-1 receptor expression (21, 90). In emerging data, semaglutide, a novel GLP-1 agonist, demonstrated superiority over older GLP-1 agonists in glycaemic control, weight reduction and cardiovascular event incidence (91, 92). The safety and efficacy of semaglutide in NAFLD is being investigated in phase II clinical trials.

Sodium-glucose transport protein-2 (SGLT-2) inhibitors impede renal glucose reabsorption, serving the dual purpose of lowering plasma glucose and enhancing caloric loss (21, 87). SGLT-2 therapy caused net weight reductions of 1.8 kg in a meta-analysis, probably as a consequence of caloric loss through glycosuria (93). SGLT-2 inhibitors are also associated with improved cardiovascular and all-cause mortality (94). In rodent models, SGLT-2 inhibition displayed protective effects on metabolic profiles and liver histology, including fibrosis (95). The translation of this benefit to human disease phenotypes is supported by sub-analyses of clinical trials showing significant reductions in transaminases with SGLT-2 therapy in T2DM (96). However, no controlled human studies of SGLT-2 inhibition in NAFLD with histological endpoints are available at present.

### Liver-specific and novel approaches

The pharmacological landscape in NAFLD therapy is evolving rapidly, yet no licensed medications for NAFLD treatment currently exist. Several promising agents are undergoing phase III development. Liver-centred treatment approaches should complement, not replace, cardiometabolic risk profile management in NAFLD patients with progressive disease. Although an ideal intervention stage is not formally defined, patients with no or mild fibrosis and low risks of fibrosis progression present a small likelihood of advancing to meaningful, liver-related outcomes. Liver-specific therapy is unlikely to produce clinically relevant, cost-effective benefits for this patient cohort. Equally, patients with decompensated NASH cirrhosis may have progressed too far for anti-fibrotic pharmacotherapy to significantly alleviate liver-related outcomes (22, 62), putting the focus of liver-specific therapies on patients with significant to advanced fibrosis (F2–F3) and compensated cirrhosis (F4). Selected patients at high risk of disease progression (e.g. NASH + T2DM with no fibrosis) may profit from liver-directed therapy following risk–benefit analysis (62).

Given the role of oxidative stress in hepatic fibrogenesis, two RCTs investigated the anti-oxidant properties of vitamin E in children and adults with NASH. In adults, vitamin E administration for 96 weeks reduced NAFLD activity score (NAS), but failed to achieve statistical significance for NASH resolution (82). In children, vitamin E therapy provided no overall benefit on liver biochemistry, steatosis or necroinflammation (97). In both trials, vitamin E therapy did not improve fibrosis significantly; however, neither trial was powered to detect clinically relevant anti-fibrotic effects (62). Long-term safety concerns regarding high-dose vitamin E use have surfaced and continue to be debated with some evidence indicating increased incidence of haemorrhagic stroke and prostate cancer (45, 98).

The dual and pan-peroxisome proliferator-activated receptor (PPAR α/δ) agonists elafibranor and lanifibranor are under active pursuit for the treatment of NASH as potent regulators of lipid metabolism (45). In animal models, elafibranor exhibited protective effects on hepatic lipid accumulation, necroinflammation and fibrosis progression (99). In the phase II GOLDEN-505 trial, elafibranor failed to achieve the primary endpoint of NASH resolution, but favoured histological improvements in more progressive disease (NAS ≥4) on *post hoc* analysis (100). A multicentre, phase III trial is currently evaluating the efficacy of elafibranor in severe steatohepatitis (NAS ≥4), although interim data outline that the trial did not meet its primary and secondary endpoints of NASH resolution and fibrosis improvement after a 72-week follow-up (https://clinicaltrials.gov/ct2/show/NCT02704403; 101). Lanifibranor has universal, agonistic action on PPA receptors (pan-PPAR) and demonstrated fibro-protective properties in rodent models (102). A phase II trial of lanifibranor safety and efficacy in NASH is ongoing (https://clinicaltrials.gov/ct2/show/NCT03008070).

Substantial evidence documents the importance of bile acids in nutritional homeostasis (62). Bile-acid-induced activation of the farnesoid X receptor (FXR) enhances insulin sensitivity and diminishes lipogenesis (103, 104). Obeticholic acid, a synthetic variant of the natural bile acid chenodeoxycholic acid, is a potent FXR ligand. The proof-of-concept, phase II FLINT trial of obeticholic acid therapy in NASH was terminated early after primary endpoints of efficacy were met during interim analysis, with 45 and 22% of patients in the intervention arm demonstrating histological improvement and NASH resolution (vs 21 and 13% in the placebo arm), respectively (105). Although not powered to evaluate fibrosis resolution or progression, the FLINT trial observed protective effects on overall fibrogenesis, demonstrating fibrosis improvement in 35% of patients in the intervention arm (vs 19% in the placebo arm) (62, 87, 105). These encouraging outcomes initiated two ongoing, multicentre, phase III trials evaluating obeticholic acid effectiveness in NASH with fibrosis (REGENERATE) and compensated NASH cirrhosis (REVERSE) (https://clinicaltrials.gov/ct2/show/NCT02548351; https://clinicaltrials.gov/ct2/show/NCT03439254). In a promising interim analysis of the REGENERATE trial, 23% of patients in the intervention arm achieved the primary endpoint of fibrosis improvement (vs 12% in the placebo group, *P* = 0.0002) (106). Main adverse effects of obeticholic acid are pruritus (~20% of patients), managed relatively successfully with topical emollients and anti-histamines in existing trials, and unfavourable serum cholesterol profiles (increased LDL, decreased HDL), generating fears of increased cardiovascular events (62). In rodent models, FXR agonism ameliorated atherosclerosis despite raising LDL levels (107). The impact of FXR agonism on cardiovascular event incidence in humans is unknown.

Other promising agents undergoing phase III development include selonsertib, which inhibits stress-induced regulators of inflammation and fibrosis, and cenicriviroc, which antagonises key drivers of monocyte recruitment and hepatic stellate cell activation (39, 45). In phase II trials, selonsertib and cenicriviroc demonstrated acceptable safety profiles and superiority over placebo in improving fibrosis (108, 109) with phase III trials in process (https://clinicaltrials.gov/ct2/show/NCT03053050; https://clinicaltrials.gov/ct2/show/NCT03028740).

## Conclusion

NAFLD is a chronic liver disease that results in a high clinical burden due to the prevalence, inherent cardiometabolic risk and potential of progressing to cirrhosis. The presence and extent of fibrosis are the most important prognostic factors in NAFLD, necessitating risk stratification of patients by fibrosis stage with non-invasive methods. A multidisciplinary approach to treatment is advised, centred on minimisation of cardiometabolic risk as the cornerstone of therapy. Despite the current lack of licensed pharmacological agents for NAFLD management, several promising agents are undergoing advanced development to complement standard management in patients with progressive disease.

## Declaration of interest

J F C has served on advisory boards and provided paid consultancy for Intercept, NovoNordisk and Alnylam. J W T has served on advisory boards and provided paid consultancy for Pfizer, AstraZeneca, Poxel, and NovoNordisk. D K has nothing to disclose.

## Funding

The authors are supported by the National Institute for Health Research (NIHR) Oxford Biomedical Research Centre (BRC). The views expressed are those of the author(s) and not necessarily those of the NHS, the NIHR or the Department of Health.
